# Supplementary material for: A fault-intrusion-tolerant system and deadline-aware algorithm for scheduling scientific workflow in the cloud
Source: PeerJ Comput Sci. 2021 Nov 2;7:e747. doi: 10.7717/peerj-cs.747 (PMC8576554; doi:10.7717/peerj-cs.747)
Supplement: Supplemental Information 6 [file peerj-cs-07-747-s006.zip › Myproject1.1/docs/org/cloudbus/cloudsim/examples/power/package-use.html]

Uses of Package org.cloudbus.cloudsim.examples.power


JavaScript is disabled on your browser.


- Overview
- Package
- Class
- Use
- Tree
- Deprecated
- Index
- Help

- Prev
- Next

- Frames
- No Frames

- All Classes

# Uses of Package org.cloudbus.cloudsim.examples.power

- Packages that use org.cloudbus.cloudsim.examples.power

  | Package | Description |
  | --- | --- |
  |  |  |
  | --- | --- |
  | org.cloudbus.cloudsim.examples.power.planetlab |  |
  | org.cloudbus.cloudsim.examples.power.random |  |
- Classes in org.cloudbus.cloudsim.examples.power used by org.cloudbus.cloudsim.examples.power.planetlab

  Class and Description

  RunnerAbstract

  The Class RunnerAbstract.
- Classes in org.cloudbus.cloudsim.examples.power used by org.cloudbus.cloudsim.examples.power.random

  Class and Description

  RunnerAbstract

  The Class RunnerAbstract.

- Overview
- Package
- Class
- Use
- Tree
- Deprecated
- Index
- Help

- Prev
- Next

- Frames
- No Frames

- All Classes
